# Drought stress in ‘Shine Muscat’ grapevine: Consequences and a novel mitigation strategy–5-aminolevulinic acid

**DOI:** 10.3389/fpls.2023.1129114

**Published:** 2023-03-15

**Authors:** Yuxian Yang, Jiaxin Xia, Xiang Fang, Haoran Jia, Xicheng Wang, Yiling Lin, Siyu Liu, Mengqing Ge, Yunfeng Pu, Jinggui Fang, Lingfei Shangguan

**Affiliations:** ^1^College of Horticulture, Nanjing Agricultural University, Nanjing, Jiangsu, China; ^2^Fruit Crop Variety Improvement and Seedling Propagation Engineering Research Center of Jiangsu Province, Nanjing, Jiangsu, China; ^3^School of Agronomy and Horticulture, Jiangsu Vocational College of Agriculture and Forestry, Jurong, Jiangsu, China; ^4^Institute of Pomology, Jiangsu Academy of Agricultural Sciences, Nanjing, Jiangsu, China; ^5^College of Life Sciences, Tarim University, Alar, Xinjiang, China

**Keywords:** grapevine, drought stress, ALA, transcriptome, metabolome, alleviation

## Abstract

Drought is a common and serious abiotic stress in viticulture, and it is urgent to select effective measures to alleviate it. The new plant growth regulator 5-aminolevulinic acid **(**ALA**)** has been utilized to alleviate abiotic stresses in agriculture in recent years, which provided a novel idea to mitigate drought stress in viticulture. The leaves of ‘Shine Muscat’ grapevine (*Vitis vinifera* L.) seedlings were treated with drought (Dro), drought plus 5-aminolevulinic acid (ALA, 50 mg/L) (Dro_ALA) and normal watering (Control) to clarify the regulatory network used by ALA to alleviate drought stress in grapevine. Physiological indicators showed that ALA could effectively reduce the accumulation of malondialdehyde (MDA) and increase the activities of peroxidase (POD) and superoxide dismutase (SOD) in grapevine leaves under drought stress. At the end of treatment (day 16), the MDA content in Dro_ALA was reduced by 27.63% compared with that in Dro, while the activities of POD and SOD reached 2.97- and 5.09-fold of those in Dro, respectively. Furthermore, ALA reduces abscisic acid by upregulating *CYP707A1*, thus, relieving the closure of stomata under drought. The chlorophyll metabolic pathway and photosynthetic system are the major pathways affected by ALA to alleviate drought. Changes in the genes of chlorophyll synthesis, including *CHLH*, *CHLD*, *POR*, and *DVR*; genes related to degradation, such as *CLH*, *SGR*, *PPH* and *PAO*; the *RCA* gene that is related to Rubisco; and the genes *AGT1* and *GDCSP* related to photorespiration form the basis of these pathways. In addition, the antioxidant system and osmotic regulation play important roles that enable ALA to maintain cell homeostasis under drought. The reduction of glutathione, ascorbic acid and betaine after the application of ALA confirmed the alleviation of drought. In summary, this study revealed the mechanism of effects of drought stress on grapevine, and the alleviating effect of ALA, which provides a new concept to alleviate drought stress in grapevine and other plants.

## 1 Introduction

With the intensification of global warming, the occurrence of drought stress will be more frequent, which will exacerbate problems with agricultural cultivation (Chaves et al., 2010). Drought seriously affects plant growth and development and reduces the growth rate of crops. This is primarily because drought affects the leaf size, stem elongation and root proliferation, stomatal movement, and water and nutrient relations of plants (Farooq et al., 2012). Under conditions of water shortage, plant leaves wither, and the edges turn yellow, which inhibits leaf development (Fanizza and Ricciardi, 2015). Furthermore, drought can result in the destruction of chloroplasts and photosynthetic machinery, which leads to a reduction in the content of chlorophyll and a significant decrease in the efficiency of plant photosynthesis (Cornic and Massacci, 1996; Reddy et al., 2004; Farooq et al., 2009; Zargar et al., 2017). The reduction in photosynthesis primarily occurs owing to stomatal or non-stomatal factors (Bota et al., 2004; Zhou et al., 2007). Moreover, drought will cause oxidative stress in plants from the cellular level, the closure of stomata, and the inhibition of photosynthetic electron transport chain, which results in the overproduction of reactive oxygen species (ROS) (Moran et al., 1994; Sade et al., 2011; Hasanuzzaman et al., 2013). Plants have evolved efficient mechanisms to adapt to drought. Stomatal closure is the first reaction to reduce transpiration under drought, which is primarily maintained by the accumulation of phytohormones, such as ABA (Pirasteh-Anosheh et al., 2016). Antioxidant and scavenging defense systems are the important bases of drought tolerance. The activities of enzymatic components, such as superoxide dismutase (SOD) and peroxidase (POD), as well as the contents of non-enzymatic components, such as glutathione (GSH), ascorbate (AsA) and α-tocopherol will change in response to oxidative stress under drought (Terzi and Kadioglu, 2006; Laxa et al., 2019). Moreover, osmotic accumulation (OA) is also a key mechanism of drought tolerance in plants, which involves the accumulation of organic solutes, such as proline, betaine (N, N, N-trimethyl glycine), soluble sugars, and sugar alcohols, and a series of inorganic salt ions, such as Ca^2+^, K^+^ and Cl^-^, to reduce the cell osmotic potential and maintain water relationship (Serraj and Sinclair, 2002; Fang and Xiong, 2015). Together, these mechanisms provide drought tolerance to plants.

Various exogenous applications of substances have been proposed to mitigate drought, which is the most common and harmful abiotic stress. For example, the combined application of 24-epibrassinolide and spermine alleviates drought-induced oxidative stress in maize (*Zea mays* L.) (Talaat et al., 2015). The application of melatonin can improve the drought tolerance of loquat (*Eriobotrya japonica* L.) seedlings (Wang et al., 2021a). The exogenous application of betaine and potassium fertilizer can improve water relationship and the yield of wheat (*Triticum aestivum* L.) under drought conditions (Raza et al., 2014). The application of exogenous glycine betaine and salicylic acid can improve the water relationship of hybrid sunflower (*Helianthus*) under conditions of water shortage (Hussain et al., 2009). As a non-toxic endogenous plant growth regulator, 5-aminolevulinic acid (ALA) has a substantial potential to alleviate abiotic stress on plants. Studies have shown that ALA can improve photosynthesis, photosystem efficiency, and the antioxidant capacity of plants (Memon et al., 2009). ALA also has the potential to alleviate some common abiotic stresses in plants, including salinity, temperature, and drought stresses (Hodgins and Van Huystee, 1986; Watanabe et al., 2000; Phung et al., 2011; Zhang et al., 2012). However, most of these studies focused on physiological aspects, and the exact mechanism of action of ALA’s mitigating effect still needs to be fully elucidated.

There is a large and growing global market for grapes (*V. vinifera*) and grape-based products. In addition to its huge economic value, as the first fruit crop whose whole genome was sequenced (Jaillon et al., 2007), many studies related to grapevine have been reported, making grapevine the model perennial fruit crop species (Gambetta et al., 2020). During the process of viticulture, vineyards are often affected by various abiotic stresses, of which drought is the most serious. Most vineyards face prolonged drought during the summer, thus, limiting grapevine growth (Lovisolo et al., 2016). ALA, a plant growth regulator that can enhance plant stress resistance, could provide a new concept to reduce drought damage in vineyards. In this study, leaves of the grapevine cultivar ‘Shine Muscat’ (‘SM’) (*Vitis labruscana* × *Vitis vinifera*) were used as research materials. The leaves were classified into control, drought treatment (Dro), and drought plus ALA (Dro_ALA) treatment. The morphological and physiological characteristics of three treated leaves were measured to examine the content of malondialdehyde (MDA) and the activities of antioxidant enzymes, such as SOD and POD. Furthermore, we combined transcriptomic and metabolome analyses to compare differentially expressed genes (DEGs) and differentially abundant metabolites (DAMs) between the ALA-treated group and the non-treated group under drought stress. The purpose of this study was to determine the protective mechanism of exogenous ALA on grapevine leaves under drought and to construct the regulatory network of drought resistance in grapevine leaves, thus, providing theoretical support for subsequent related studies.

## 2 Materials and methods

### 2.1 Plant materials, treatments, and sampling

Two-year-old ‘SM’ grapevines were grown in the heliogreenhouse (relative humidity of ~85% and a temperature regime of 25 °C day/15 °C night) of the Baima Teaching and Research Base of Nanjing Agricultural University, Nanjing, China (31°36′36′′ N, 119°10′48′′ E). They were used as the plant material for this study. Equal proportions of perlite, peat and horticultural vermiculite (1:1:1, v/v/v) were used to grow the grape seedlings. First, each pot of seedling soil was flooded with water and then allowed to dry out. Ten days after the watering had stopped was defined as 0 d, and the soil water content was below 9% at this time. The leaves in the Dro_ALA treatment was sprayed with 50 mg·L^-1^ ALA on both sides until the leaf surface was soaked once every three days from 0 d. The same amount of distilled water was sprayed on the leaves for the Dro treatment. The control plants were watered daily to field capacity. Leaves were collected at 0, 2, 4, 6, 8, 10, 12, 14, and 16 days, and phenotypic observations and physiological indices were measured.

### 2.2 Measurement of physiological and biochemical responses

The content of MDA and activities of POD and SOD were measured as previously described (Beauchamp and Fridovich, 1971; Zheng and Van Huystee, 1992; Tewari et al., 2002). Cellulose acetate glue was applied to the leaf surface to form a thin layer, and the glue was dried into a thin film, which was imaged in an automatic positive fluorescence microscope (DM6 B; Leica Microsystems, Wetzlar, Germany). The stomatal apertures were also measured using this system. The soil water content (WC) was determined by the gravimetric method (Little et al., 1998). Each sample had three biological replicates.

### 2.3 Transcriptome and metabolome sequencing and multi-omics analysis

Transcriptome sequencing was performed on samples from the 16^th^ day of the treatments. Total RNA was extracted and sequenced as described by Huang et al. (2020). Reads obtained from the sequencing machines included raw reads that contained adapters or low-quality bases that would affect the following assembly and analysis. Thus, to obtain high quality clean reads, the reads were further filtered by FASTP version 0.18.0 (Chen et al., 2018). The parameters were as follows: (1) removal of the reads that contained adapters; (2) removal of the reads that contained > 10% of unknown nucleotides (N); and (3) removal of low quality reads that contained > 50% of low quality (Q-value ≤ 20) bases. |log_2_ fold change (FC)| ≥ 1.0 and adjusted *P*-value (*p*_adj_) < 0.05 were used as the screening criteria for DEGs. Metabolome sequencing was determined by liquid chromatography-mass spectrometry (LC-MS) based on high-performance liquid chromatography (HPLC) (UltiMate 3000; Thermo Fisher Scientific, Waltham, MA, USA) and mass spectrometry (Q Exactive; Thermo Fisher Scientific). As for DAMs, the variable importance in the projection (VIP) value of a multivariate statistical analysis of orthogonal projections to latent structure discriminant analysis (OPLS-DA) and the *t*-test *P*-values of univariate statistical analysis were combined to screen the metabolites with significant differences between the different comparison groups (VIP > 1 and *P*-value < 0.05) (Saccenti et al., 2014). The transcriptomic and metabolomic data were integrated by a two-way orthogonal partial least squares (O2PLS) analysis (Bylesjö et al., 2007) using the Omics PLS package. Pearson correlation coefficients were calculated to integrate the metabolome and transcriptome data. Gene and metabolite pairs were ranked in the descending order of absolute correlation coefficients. The top 50 genes and metabolites were selected for heatmap analysis using pheatmap packages in the R project. Additionally, the top 250 pairs of genes and metabolites (with an absolute Pearson correlation > 0.5) were subjected to metabolite-transcript network analysis using igraph packages in the R project (Csardi and Nepusz, 2006). Cytoscape software (version 3.6.1) was used to visualize the pairs of genes and metabolites.

### 2.4 Analysis of the experimental results

The DEGs and DAMs between the groups were measured to analyze the major response of the ‘SM’ grapevine to drought stress and the mitigating effects of ALA. Comparison groups were established as follows: (I) DEGs and DAMs between the control versus Dro (control *vs*. Dro) comparison group were analyzed to determine the effect of drought stress on grapevine. (II) The control versus Dro_ALA (control *vs*. Dro_ALA) comparison group was used to analyze the difference between the ALA treatment after drought stress and the control treatment, and (III) The alleviating effect of ALA on drought stress was determined by analyzing the DEGs and DAMs between the Dro and Dro_ALA (Dro *vs.* Dro_ALA) treatments.

### 2.5 Statistical analysis

TBtools software (version 1.0692) and MapMan software (version 3.6.0) were used to analyze the functions of DEGs (Thimm et al., 2004). The data were expressed as the mean ± standard deviation (SD). A one-way analysis of variance (ANOVA) was used to analyze the data. Tukey’s multi-range test was conducted using GraphPad Prism software (version 8.0.2) (GraphPad Software, San Diego, CA, USA) to determine significant differences between and within groups at P < 0.05 (Saccenti et al., 2014).

### 2.6 Validation of RNA-seq using RT-qPCR

Ten DEGs were randomly selected for quantitative reverse transcription PCR (RT-qPCR) analysis to verify the precision and repetitiveness of the transcriptome analytical results. Purified RNA samples were reverse-transcribed using the Revert Aid™ First-Strand cDNA Synthesis Kit (Fermentas, Glen Burnie, MD, USA) following the manufacturer’s instructions. Real-time quantitative reverse transcription PCR (qRT-PCR) was performed using a Quantagene q225 system (Kubo Tech, Beijing, China). Specific primers were designed using Primer Premier 5 software (**Table S1**; Premier Biosoft, Palo Alto, CA, USA). The *V. vinifera* actin gene (*VvActin*, AB073011) was used as the internal control gene. Each sample had three replicates. Gene expression was calculated using the 2^-ΔΔCT^ method (Livak and Schmittgen, 2001).

## 3 Results

### 3.1 ALA can alleviate drought stress on grapevine leaves by increasing the activity of antioxidant enzymes

**Figure 1:**
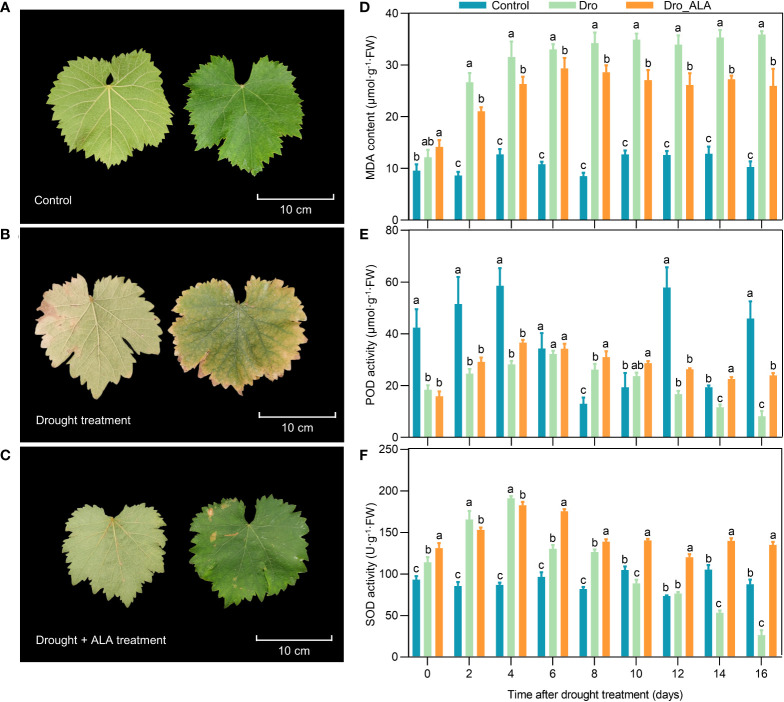
Leaf phenotype observation and physiological index determination **(A)** Leaf phenotype of control on the 16^th^ day. **(B)** Leaf phenotype of drought treatment (Dro) on the 16^th^ day. **(C)** Leaf phenotype of drought plus 5-aminolevulinic acid treatment (Dro_ALA) on the 16^th^ day. **(D)** Malondialdehyde (MDA) content changes with time under control, Dro and Dro_ALA groups. **(E)** Peroxidase (POD) activity changes with time under control, Dro and Dro_ALA groups. **(F)** Superoxide dismutase (SOD) activity changes with time under control, Dro and Dro_ALA groups. In **Figure 1C**, the left side of each picture shows the bottom surface of the leaf, and the right side shows the top side of the leaf. Different letters in a column indicate significance of difference between treatments (P ≤ 0.05).

The leaf morphology of ‘SM’ grapevine seedlings was significantly affected by drought treatment. Under drought conditions, the leaves first wilted on the fourth day, and they were significantly withered and became yellowed on the tenth and the 16^th^ days, respectively. The leaves were already producing significant drought symptoms. However, the Dro_ALA leaves showed only mild symptoms of drought stress (**Figures 1A-C** and **S1**). The drought-induced MDA accumulation in grapevine leaves was significantly reduced by the ALA treatment, and on the tenth day, the MDA content in Dro_ALA leaves was 28.94% lower than that in the Dro leaves (**Figure 1D** and **Table S2**). The POD activity in the Dro treatment kept increasing until the sixth day, and then it gradually decreased, while the POD activity in Dro_ALA was higher than that in the Dro treatment (**Figure 1E** and **Table S2**). The activity of SOD increased first and then decreased under drought. In the Dro_ALA treatment, the SOD activity showed the same trend as that in the Dro, which peaked on the fourth day and then decreased. However, the SOD activity in the ALA treatment group decreased more slowly (**Figure 1F** and **Table S2**). These results indicate that exogenous ALA may effectively prevent ‘SM’ grapevine seedlings from prolonged drought by increasing the activity of antioxidant enzymes. Moreover, on the 16^th^ day, the symptoms of drought stress were evident, so the leaf samples of the control, Dro and Dro_ALA plants from the 16^th^ day of treatment were collected for transcriptome and metabolome sequencing.

### 3.2 Mechanisms of the response of grapevine leaves to drought and ALA

**Figure 2:**
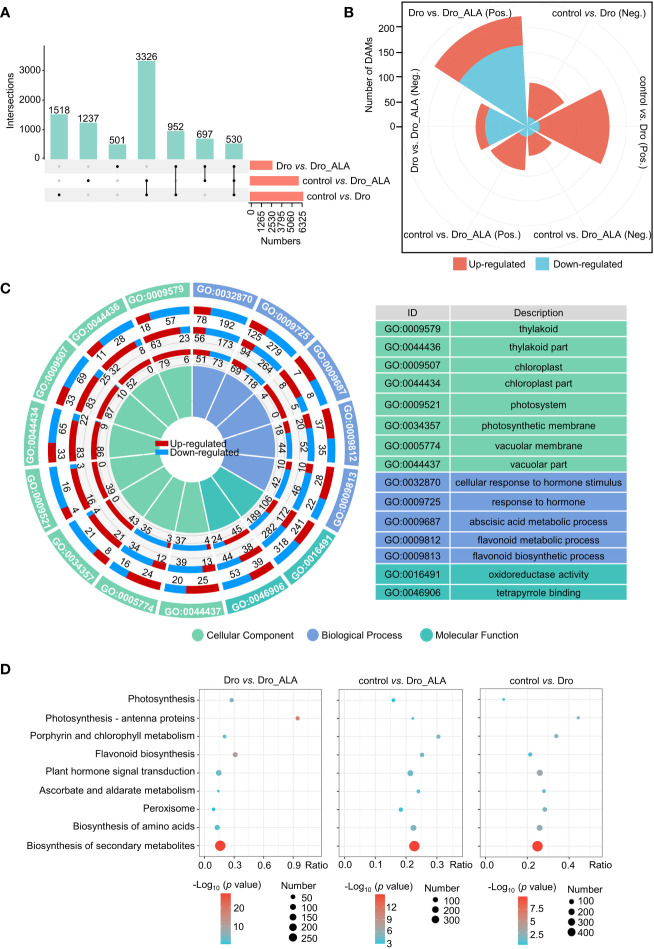
Functional annotations of total DEGs and DAMs. **(A)** Upset plot of DEGs between control and drought treatment (control *vs.* Dro); control and drought plus 5-aminolevulinic acid treatment (control *vs.* Dro_ALA); drought and drought and 5-aminolevulinic acid treatment (Dro *vs.* Dro_ALA). **(B)** Statistics of significant difference of DAMs between control *vs.* Dro, control *vs.* Dro_ALA, and Dro *vs.* Dro_ALA. The threshold of significant difference was VIP≥1 and T-test P<0.05.(Pos. means positive and Neg. means negative) **(C)** Information about key GO terms of control *vs.* Dro, control *vs.* Dro_ALA, and Dro *vs.* Dro_ALA. The first circle represents the GO ID, the second circle represents the DEGs between control *vs.* Dro, the third circle for control *vs.* Dro_ALA, and the fourth for Dro *vs.* Dro_ALA. Red and blue indicate up-regulated and down-regulated DEGs, respectively. **(D)** Information about key pathways in the KEGG enrichment pathways of control *vs.* Dro, control *vs.* Dro_ALA, and Dro *vs.* Dro_ALA.

The control, Dro, and Dro_ALA samples were subjected to RNA-seq and metabolome analyses (**Table S3**). A total of 6,326, 5,790, and 2,680 DEGs (**Figure 2A**) and 255, 147, and 327 DAMs (**Figure 2B**) were detected between the three comparison groups (control *vs.* Dro; control *vs.* Dro_ALA; and Dro *vs.* Dro_ALA). The intersection of different numbers of DEGs in each comparison is shown in **Figure 2A**. We demonstrated the correlation of each sample in **Figures S2** and **S3** and **Tables S3** and **S4**, and we found that the DEGs significantly correlated with the DAMs (PCC) > 0.8 (**Figure S4**). Details of the DEGs and DAMs are shown in **Tables S5-S20**. A Gene Ontology (GO) analysis indicated that ‘thylakoid’, ‘thylakoid part’, ‘chloroplast’, and ‘chloroplast part’ were enriched in the control *vs*. Dro and control *vs*. Dro_ALA comparison group. This result suggests that drought could affect chlorophyll anabolism. In the meantime, the enrichment of ‘photosystem’, ‘photosynthesis membrane’ in the control *vs*. Dro and control *vs*. Dro_ALA indicated that photosynthesis was affected under drought stress (**Figures 2C** and **S5**; **Table S17**). Consistent with this, the Kyoto Encyclopedia of Genes and Genomes (KEGG) enrichment analysis of the DEGs revealed that ‘porphyrin and chlorophyll metabolism’, ‘photosynthesis-antenna proteins,’ and ‘photosynthesis’ were significantly enriched in the control *vs.* Dro comparison group (**Figures 2D** and **S6**; **Table S18**). In the control *vs*. Dro_ALA comparison group, most of the DEGs in GO terms that were related to chlorophyll metabolism and photosynthesis were upregulated (**Figures 2C** and **S5**; **Table S17**), indicating that ALA could play a positive role in mitigating the inhibition of photosynthesis in grapevine under drought. Furthermore, phytohormones play a role in drought tolerance, and ALA alleviates drought in grapevine. ‘Response to hormone,’ ‘cellular response to hormone stimulus’ in GO and ‘plant hormone signal transduction’ in KEGG were significantly enriched. Moreover, the enrichment of molecular function, such as ‘oxidoreductase activity’ in GO and ‘peroxisome’ and ‘ascorbate and aldarate metabolism’ in the KEGG analysis in each comparison group showed that antioxidant systems also play a vital role in drought resistance in grapevine and the process of alleviating drought stress by ALA (**Figures 2D** and **S6**; **Table S18**). The KEGG analysis of the metabolome also detected enriched terms, such as the ‘biosynthesis of secondary metabolism’ (**Figure S7** and **Table S19**). Moreover, qRT-PCR was used to validate the reliability of RNA-seq (**Figure S8**; **Table S1**). The function of DEGs was analyzed using MapMan software as shown in **Tables S20** and **S21**.

### 3.3 ALA inhibits stomatal closure induced by abscisic acid in grapevine leaves under drought stress

**Figure 3:**
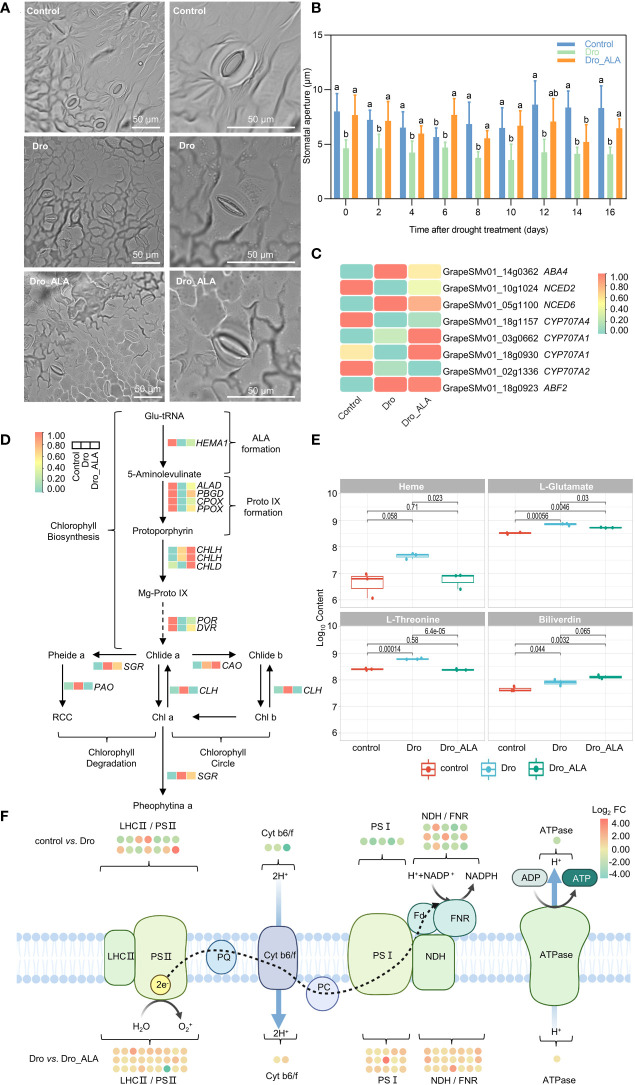
Stomatal movement, chlorophyll metabolism and photosynthesis in grapevine leaves under drought stress and drought plus ALA treatment. **(A)** Stomatal aperture observation of control, drought treatment (Dro), and drought plus 5-aminolevulinic acid treatment (Dro_ALA) on the 16^th^ day (via upright fluorescent microscope). **(B)** Variation of stomatal aperture with drought treatment time. **(C)** Key DEGs related to phytohormones action between control *vs.* Dro, control *vs.* Dro_ALA, and Dro *vs.* Dro_ALA. **(D)** Pathway of chlorophyll metabolism and key genes in grapevine under drought stress and drought plus 5-aminolevulinic acid treatment. Arrows (↑) and (↓) indicate up-regulation and down-regulation, respective. Red indicates the control vs. Dro comparison group and blue indicates the Dro vs. Dro_ALA comparison group. **(E)** Key metabolites in chlorophyll metabolism pathway under control, Dro and Dro_ALA treatments. **(F)** Effects of drought and drought plus 5-aminolevulinic acid on photosynthesis. Each dots represents a DEG, green and red indicate up-regulation and down-regulation, respectively. Different letters indicate significant differences among experimental groups (p < 0.05).

In this study, the grapevine stomatal aperture was found to be significantly reduced compared with the control under drought. Although it was still smaller than the control, the stomatal aperture increased after the ALA treatment (**Figures 3A, B**; **Table S22**). In the 16^th^ day, the stomatal aperture of control was 103.7% larger than that of Dro, while Dro_ALA was 58.4% larger than that of Dro. (**Figures 3A, B**, **Table S22**). It is a consensus that ABA induces plants to close their stomata to resist stress under drought (Bray, 1997; Buckley, 2019; Ilyas et al., 2020). The DEGs of factors associated with ABA synthase, such as neoxanthin synthase (*ABA4*, |log_2_ FC| = 1.26) and 9-*cis*-epoxycarotenoid dioxygenase6 (*NCED*6, |log_2_ FC| = 3.35) were significantly upregulated in the control *vs.* Dro comparison. Simultaneously, in the Dro treatment, the genes of factors related to the decomposition of ABA, such as abscisic acid 8’-hydroxylase (*CYP707A1*, *CYP707A2*, and *CYP707A4*), were expressed at lower levels than those in the control. This led to the accumulation of endogenous ABA under drought. In the metabolome, (S)-abscisic acid (POS_M265T406; Log_10_ content from 8.71 to 8.82) increased after drought treatment, which was consistent with the results described above. It is worth noting that the upregulation of *ABF2* (|log_2_ FC| = 1.48), an ABRE-binding bZIP factor, was also detected as upregulated in the control *vs.* Dro comparison, which confirmed the accumulation of ABA (**Figure 3C**). The downregulation of some genes, such as *ABA4* (|log_2_ FC| = 0.53), *NCED6* (|log_2_ FC| = 0.41), and *ABF2* (|log_2_ FC| = 0.04), were detected in the Dro *vs.* Dro_ALA comparison. Moreover, *CYP707A1* (|log_2_ FC| = 1.79) was significantly upregulated after the application of ALA. In the metabolome, the content of (S)-abscisic acid in Dro_ALA was lower than that of Dro (Log_10_ content from 8.82 to 8.70). These findings suggest that ALA could primarily reduce the accumulation of ABA by accelerating the degradation of ABA, thus, resulting in the re-enlargement of stomatal aperture.

### 3.4 ALA alleviates the inhibition of photosynthesis by drought on grapevine leaves

Phenotypic observations showed that the leaves suffered from severe chlorosis under drought (**Figures 1** and **S1**), which could be related to the degradation of chlorophyll. In the control *vs.* Dro comparison, the level of expression of *glutamyl-tRNA reductase* (*HEMA1*) was downregulated by 31.2%, which led to the inhibition of endogenous ALA formation (**Figure 3D**; **Table S25**). Transcripts involved in the factors of protoporphyrin synthesis, such as porphobilinogen synthase (*ALAD*), porphobilinogen deaminase (*PBGD*), coproporphyrinogen III oxidase (*CPOX*), and protoporphyrinogen oxidase (*PPOX*), were all maintained at low levels of expression under drought conditions (**Figure 3D**; **Table S25**). The genes for protochlorophyllide oxidoreductase (*POR*) and divinyl chlorophyllide a 8-vinyl-reductase (*DVR*) were also downregulated in the control *vs.* Dro comparison (**Figure 3D**; **Table S25**). These results indicate that chlorophyll biosynthesis is inhibited under drought stress. In contrast, chlorophyll conjugation and degradation genes, such as the genes that encode chlorophyllide a oxygenase (*CAO*), chlorophyllase (*CLH*), magnesium dechelatase (*SGR*), and pheophorbide a oxygenase (*PAO*), were all downregulated in the control *vs.* Dro comparison. Changes in these genes led to a reduction in chlorophyll accumulation in grapevine leaves under drought (**Figure 3D**; **Table S25**). In porphyrin metabolism, an increase of L-glutamate (Log_10_ content from 8.52 to 8.85) and L-threonine (Log_10_ content from 8.52 to 8.85) were detected (**Figures 3E** and **S9**).

In this study, the application of ALA upregulated the levels of expression of genes related to chlorophyll synthesis and inhibited the levels of expression of the genes related to degradation. Compared with Dro, the levels of expression of genes, such as *HEMA1*, *ALAD*, *PBGD*, *CPOX*, and *PPOX*, all increased in Dro_ALA. Simultaneously, in the Dro *vs.* Dro_ALA comparison, *CHLH*, *CHLD*, *POR*, and *DVR* were all upregulated, indicating that the inhibition of chlorophyll synthesis was relieved. Moreover, the downregulation of *CLH*, *SGR*, *PPH*, and *PAO* was detected in Dro *vs.* Dro_ALA, which resulted in the inhibition of chlorophyll degradation (**Figure 3D**; **Table S25**). In the metabolome, the contents of L-glutamate (Log_10_ content from 8.85 to 8.72) and biliverdin (Log_10_ content from 7.91 to 8.11) increased, while those of heme (Log_10_ content from 7.67 to 6.80) and L-threonine (Log_10_ content from 8.79 to 8.39) decreased (**Figures 3E** and **S9**). These combined effects resulted in an increase in chlorophyll synthesis and a decrease in chlorophyll degradation after the application of ALA.

The photosynthetic electron transport chains were also inhibited under drought. A comparison of Dro to the Control showed that approximately 66% DEGs that are involved in photosynthesis were downregulated (**Figure 3F**; **Table S24**). Eight of the 14 DEGS related to photosystem II were downregulated in the Control *vs.* Dro comparison. Most of them were associated with the LHC-II complex and PSII assembly and maintenance. The functions of cytochrome b6/f complex, photosystem I, and ATP synthase complex were also inhibited, and the corresponding three, five and one DEGs detected in the control *vs.* Dro, respectively, were all downregulated. Furthermore, seven of the DEGs related to the NADH dehydrogenases were downregulated in the control *vs.* Dro. As a vital enzyme in the Calvin cycle, the activity of Rubisco was also inhibited under drought. This could be owing to the downregulation of DEGs related to the CPN20 auxiliary co-chaperone (*CPN20*, |log_2_FC| = 7.21) and BSD2 assembly factor (*BSD2*). The downregulation of genes described above resulted in a significant decrease in the photosynthetic efficiency under drought stress. Moreover, the DEGs related to glycolate oxidase (*GLO1*), glutamate-glyoxylate transaminase (*GGAT2*), and serine-glyoxylate transaminase (*AGT1*) were all maintained at high levels of expression in Dro, which resulted in enhanced photorespiration (**Table S20**). This could further weaken photosynthesis.

In the Dro *vs.* Dro_ALA comparison, only two of 82 DEGs related to photosynthesis were downregulated, and most of them were also downregulated in the control *vs.* Dro treatment (**Figure 3F**; **Table S24**). Changes in the DEGs associated with PSII (26 upregulated and one downregulated), cytb6/f complex (four upregulated), PSI (15 upregulated), ferredoxin electron carrier (one upregulated), ferredoxin NADP^+^ oxidoreductase (FNR) (three upregulated), NDH complex (17 upregulated) and ATP synthase complex (one upregulated) were detected (**Table S24**). A significant upregulation of the DEGs related to the activity, assembly, and regulation of Rubisco, such as *CPN60B4*, *RBCX1*, *RBCX2*, *BSD2*, and *RCA*, was detected after ALA treatment, indicating that the inhibition of Rubisco under drought was relieved. Moreover, treatment with ALA led to upregulation of the genes that encoded serine-glyoxylate transaminase (*AGT1*), glycine dehydrogenase component P-protein of the glycine cleavage system (*GDCSP*), aminomethyl transferase component T-protein of the glycine cleavage system (*GDCST*), and lipoamide-containing component H-protein of the glycine cleavage system (*GDCSH*), which indicated that ALA reduces photorespiration, and thus, alleviates drought stress (**Table S21**).

### 3.5 ALA alleviates oxidative stress in grapevine leaves

**Figure 4:**
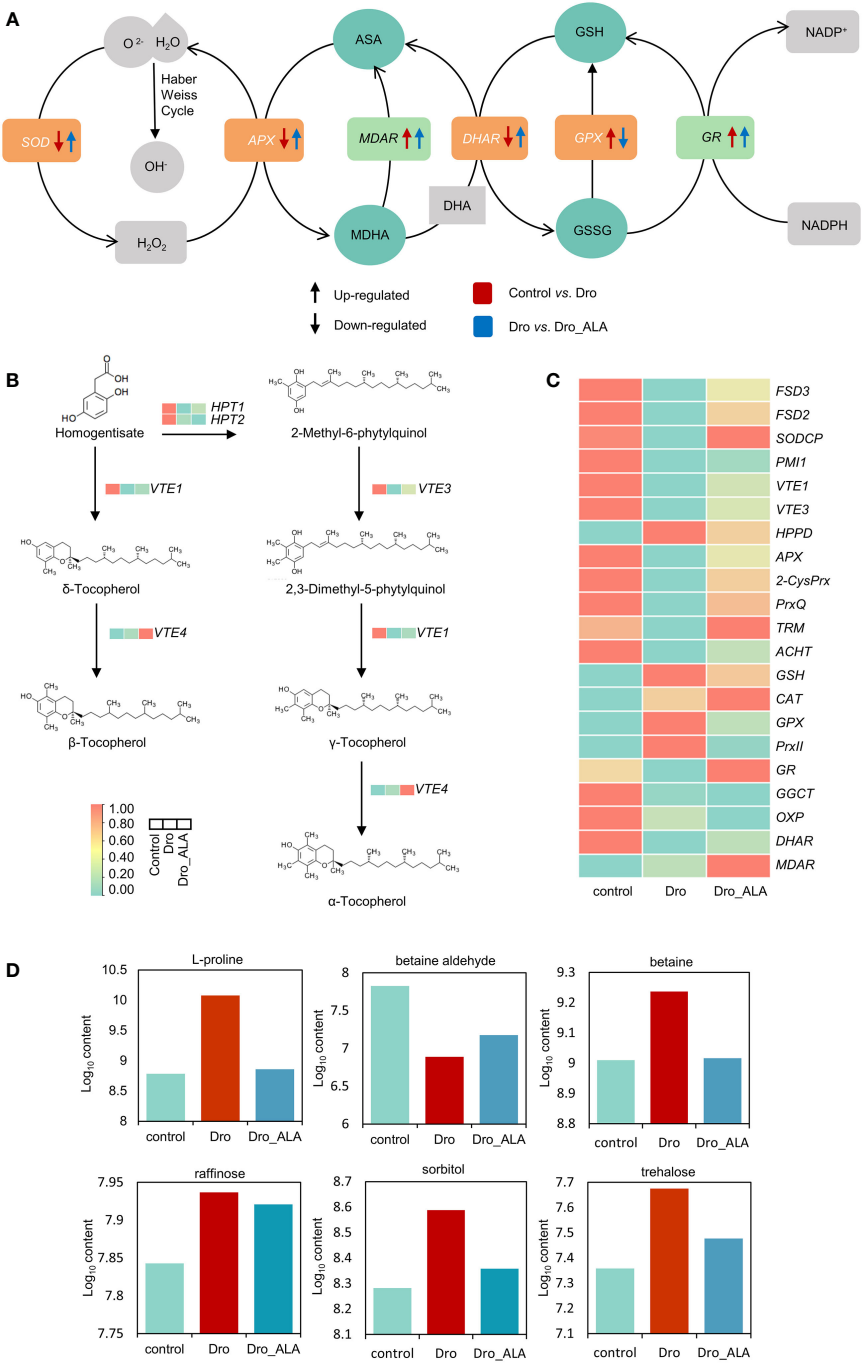
Cellular homeostasis under drought treatment and drought plus ALA treatment. **(A)** AsA-GSH cycle of grapevine leaves under drought stress and drought plus 5-aminolevulinic acid treatments, Arrows (↑) and (↓) represent up-regulation and down-regulation, respectively. Red and blue represent control *vs*. Dro and Dro *vs*. Dro_ALA groups, respectively. **(B)** Biosynthesis of tocopherol in grapevine leaves under control, under drought stress and drought plus 5-aminolevulinic acid treatments. Red and green represent higher and lower expression, respectively. **(C)** Information of DEGs related to antioxidant system of grapevine in control, Dro and Dro _ALA. **(D)** Information of important DAMs related to osmotic regulation and autophagy of grapevine in control, Dro and Dro _ALA. (Dro represents drought treatment; Dro_ALA represents drought plus 5-aminolevulinic acid treatment).

In this study, the upregulation of NADPH oxidase (*RBOHA*, 21.52-29.21 fragments per kilobase of transcripts per million mapped reads [FPKM]) related to ROS generation was detected in the control *vs*. Dro comparison, indicating that there is a mass production of ROS under drought stress (**Table S26**). Drought inhibited the expression of some genes related to antioxidant scavenging. For example, the DEGs related to iron superoxide dismutase (*FSD3* and *FSD2*) and copper/zinc superoxide dismutase (*SODCP*) were significantly downregulated (**Figure 4C**; **Table S26**). The genes of some low-molecular weight scavengers, such as phosphomannose isomerase (*PMI*), related to the biosynthesis of ascorbate also showed a similar trend. The α-, β-, γ-, and δ-forms of tocopherol are active antioxidants that are primarily located in chloroplast membranes where they detoxifying singlet oxygen and lipid peroxy radicals (Munné-Bosch, 2005). In the control *vs.* Dro comparison group, the genes related to tocopherol biosynthesis, such as *VTE1* and *VTE3*, were both downregulated (**Figure 4B** and **Table S26**). As expected, the levels of α-tocopherol and ascorbate were indeed decreased in the metabolome (**Figure S10**; **Table S27**). In the ascorbate-glutathione cycle, the level of expression of the gene related to ascorbate peroxidase (*APX*, 20.97 to 6.46 FPKM) was also suppressed. The chloroplast redox homeostasis was disrupted under drought stress, which could inhibit the photosynthesis of grapevine leaves even further. DEGs related to typical 2-Cys peroxiredoxin (*2-CysPrx*), atypical 2-Cys peroxiredoxin (*PrxQ*), M-type thioredoxin (*TRM*) and atypical thioredoxin (*ACHT*) maintained low levels of expression. Simultaneously, the levels of monodehydroascorbate reductase (*MDHAR*, 29.26 to 31.50 FPKM) in the ascorbic acid-glutathione (AsA-GSH) cycle and γ-glutamyl cysteine ligase (GSH) associated with glutathione biosynthesis were upregulated. The DEGs related to catalase (*CAT*, 305.9 to 747.97 FPKM), glutathione peroxidase (*GPX6*, 238.037 to 429.523 FPKM), and type-2 peroxiredoxin (*PrxII*) showed the same trend (**Figure 4C**; **Table S26**). In contrast, the DEGs associated with glutathione degradation, such as glutathione reductase (*GR*, 92.6 to 77.30 FPKM), γ-glutamyl cyclotransferase (*GGCT*), oxoprolinase (*OXP*), and dehydroascorbic acid reductase (*DHAR*, 42.34 to 26.08 FPKM), were downregulated (**Figure 4C** and **Table S26**). This could indicate that the glutathione content was increased to remove the ROS. Together, changes in the genes described above led to the accumulation of intracellular ROS, which caused drought oxidative stress. The metabolome data were consistent with the results described above and showed that the contents of glutathione, L-cysteine and oxidized glutathione increased (**Figure S10**; **Table S27**).

The application of ALA inhibited the generation of ROS, *RBOHA*, *RBOHB*, and *RBOHC*, which were all downregulated in the Dro *vs*. Dro_ALA comparison group (**Table S26**). ALA could enhance the function of ROS scavenging system in grapevine under drought stress. A comparison of Dro with Dro_ALA showed that *FSD3*, *FSD2* and *SODCP* restored upregulation (**Figure 4C**; **Table S26**). In addition, the upregulation of *VTE1* and *VTE3* indicated that tocopherol was resynthesized, and the content of α-tocopherol increased in the metabolome (**Figure S10** and **Table S27**). In the AsA-GHS cycle, ascorbate peroxidase (*APX*) and glutathione peroxidase (*GPX8*), and the genes related to type-2 peroxiredoxin (*PrxII*) all tended to be downregulated after application with ALA (**Figure 4C**; **Table S26**). This suggests that the drought stress was easing. The effect of ALA on photosynthesis was shown in the recovery of chloroplast redox homeostasis, and the genes of atypical 2-Cys peroxiredoxin (*PrxQ*), M-type thioredoxin (*TRM1*), and atypical thioredoxin (*ACHT*) were significantly upregulated. These processes reduce the production of ROS and ease the cellular damage caused by drought stress. In the metabolome of the Dro *vs*. Dro_ALA comparison group, the contents of glutathione and ascorbate decreased (**Figure S10**; **Table S27**). Changes in the genes and metabolites described above suggest that ALA does alleviate the oxidative stress caused by drought.

### 3.6 Osmotic regulation under drought stress

OA has often been considered to be a key mechanism of the resistance of plants to drought stress (Serraj and Sinclair, 2002). Proline has long been thought to accumulate in plants that experience water restriction (Verslues and Sharma, 2010). Pyrroline-5-carboxylate synthetase (*PRO2*, 52.19 to 202.07 FPKM), ornithine aminotransferase (*OAT*, 15.88 to 60.90 FPKM) and PHR1 transcription factor (*PHR1*, 3.61 to 13.42 FPKM) involved in the regulation of proline synthesis were significantly upregulated in the Control *vs*. Dro comparison group (**Table S28**). Consistent with this result, increased levels of L-proline were detected in the metabolome (**Figure 4D**; **Table S29**). As a quaternary ammonium compound, betaine (N, N, N-trimethyl glycine) is also a vital solute involved in osmotic regulation. Betaine aldehyde dehydrogenase (BADH) catalyzes the conversion of betaine aldehyde to betaine (Weretilnyk and Hanson, 1990). *BADH4* associated with BADH tended to be upregulated after drought stress, and this corresponded to a significant decrease in the contents of betaine aldehyde and an increase in the content of betaine in the metabolome (**Figure 4D**; **Table S29**). The contents of some soluble sugars and sugar alcohol also changed. For example, trehalose, raffinose, and sorbitol were all elevated under water deficiency (**Figure 4D**; **Table S29**). A sharp decrease in proline content (Log_10_ content from 10.08 to 8.86) was detected in the metabolome of Dro *vs*. Dro_ALA comparison group, which was caused by the downregulation of *PRO2* (202.07 to 101.14 FPKM), *OAT* (60.90 to 33.71 FPKM), and *PHR1* (13.42 to 7.64 FPKM). In this study, *BADH4* was downregulated, which resulted in an increase in betaine aldehyde, and a decrease in betaine (**Figure 4D**; **Tables S28** and **29**). The decrease in the contents of soluble sugars and sugar alcohols also indicated that drought stress tended to be alleviated after the application of ALA. The contents of raffinose, sorbitol, and trehalose all decreased in the Dro *vs*. Dro_ALA comparison group (**Figure 4D**; **Table S29**). These results indicate that the cell homeostasis tends to moderate.

## 4 Discussion

### 4.1 ALA uses multiple synergistic mechanisms to alleviate drought stress

Exogenous plant growth regulators are widely used to alleviate drought stress in grapevine. For example, exogenous melatonin can improve the resistance of wine grape ‘Riesling’ seedlings to water deficiency by alleviating PSII damage and protecting the chloroplasts (Meng et al., 2014). The application of exogenous 24-epibrassinolide (EBR) has also been shown to alleviate the inhibition of drought stress on grape photosynthesis by increasing the content of chlorophyll and alleviating stomatal and non-stomatal limitations on photosynthetic performance (Wang et al., 2015). Consistent with this, ALA alleviates the stomatal closure caused by drought and thus, moderates the decrease in photosynthesis to some extent (**Figures 3A, B**; **Table S22**). As a precursor of chlorophyll biosynthesis, ALA can directly increase chlorophyll synthesis and inhibit chlorophyll degradation under drought stress (**Figure 3D**). The inhibition of photosynthetic electron transport chain was also relieved by ALA under drought (**Figure 3F**). This also impacted the genes related to Rubisco and alleviated photorespiration (**Table S24**). Exogenous growth regulators can also alleviate the imbalance of grapevine cell homeostasis caused by drought stress. Strigolactones upregulate the antioxidant enzyme genes *CAT1* and *APX6* to alleviate drought stress in ‘Cabernet Sauvignon’ seedlings (Wang et al., 2021b). The application of ABA increases the contents of proline and soluble sugars and the activities of SOD and POD in ‘Red Globe’ grape. ALA mitigated drought in a manner similar to that of the study described above. In this study, the application of exogenous ALA reduced the contents of MDA and inhibited the production of ROS in ‘SM’ seedlings under drought, activated antioxidant system by upregulating *FSD* and *SODCP* and increased the content of α-tocopherol and other non-enzymatic antioxidant scavengers (**Figure 4C, D**). Therefore, this study provides a novel idea for ALA to alleviate grapevine drought stress.

### 4.2 Mitigating effects of ALA on grapevine photosynthesis under drought stress

The formation of ALA is a rate-limiting step in chlorophyll biosynthesis (Beale, 1990), and many studies (Kosar et al., 2015; Wang et al., 2018; Rasheed et al., 2020) have shown that ALA enhances the resistance of plants to drought stress by enhancing photosynthesis. For example, pretreatment with ALA increases stomatal conductance and thus, stabilizes photosynthesis in wheat under drought. Rasheed et al. (2020) demonstrated that ALA alleviates the drought stress of sunflower (*H. annuus* L.) by protecting chlorophyll from degradation. The exogenous application of ALA alleviates drought stress by enhancing the chlorophyll pigments of spring wheat seedlings. However, these studies are based on physiological indicators, and the changes in genes and metabolites during drought were unclear. In our study, ALA treatment significantly increased the stomatal aperture during drought (**Figure 3A, B**; **Table S22**). ALA leads to an increase in the level of expression of *CYP707A1* under drought, which reduces the content of ABA and leads to further stomatal opening (**Figure 3C**). In addition, ROS are important signals that regulate stomatal closure (Song et al., 2014), our study shows that the antioxidant system reduces ROS production after ALA application, which may also lead to stomatal reopening, but the specific mechanism is still unclear. GSH plays a role in stomatal movement, it is generally believed that increased GSH content leads to stomatal opening. For example, studies on the negative regulation of glutathione in *Arabidopsi*s thaliana on stomatal closure induced by methyl jasmonate have been reported (Akter et al., 2013). In this study, although we detected stomatal opening caused by ALA, GSH content decreased after application of ALA, which was inconsistent with the above study. We hypothesized that GSH did not play a major role in ROS reduction and stomatal opening after ALA application. Although the inhibition of water lost by transpiration that is limited by the closure of stomata induced by ABA is an important mechanism to improve drought tolerance in plants (Li et al., 2006), a previous study indicated that the closure of stomata induced by ABA does not increase plant sensitivity to drought stress (An et al., 2016). In this study, we hypothesized that during mild drought, the positive effect of stomatal opening and enhancing photosynthesis by ALA was greater than the negative effect of water loss caused by transpiration. Nevertheless, the molecular mechanisms behind the paradox between the prohibition of stomatal opening and an enhancement of drought tolerance merits further study. As for non-stomatal factors, first, after the application of ALA, genes related to chlorophyll synthesis, such as *HEMA1*, *ALAD*, PBGD, *CPOX*, and *PPOX*, were significantly upregulated, while the levels of expression of *CLH*, *SGR*, *PPH*, and *PAO* were downregulated, which resulted in the inhibition of chlorophyll degradation (**Figure 3D** and **Table S25**). Consistent with the DEGs, the contents of L-glutamate and biliverdin increased, while those of heme and L-threonine decreased. These changes in the genes and metabolites together serve as the basis for ALA to alleviate the loss of chlorophyll in grape leaves under drought (**Figures 3D, E** and **S9**). It is worth mentioning that the relation between porphyrin metabolism and the content of L-glutamate and L-threonine is not well followed. Our data support the results that the above metabolites are related to porphyrin and chlorophyll metabolism, the specific mechanisms are still meriting further study. Furthermore, Cai et al. (2020) found that spraying 10 mg/L ALA on the leaves alleviated the reduction in the activities of PSI and PSII reaction centers induced by PEG 6000, electron transport activity, and photosynthetic performance indices in strawberry (*Fragaria × annanasa* Duch. cv. ‘Benihoppe’). The transcriptomic data showed that ALA upregulated the genes related to PSII and those related to the cytochrome B6/F complex in plants that had been subjected to drought stress (**Figure 3F**), which was consistent with our conclusions above. Notably, ALA treatment alleviates drought stress by upregulating the levels of expression of *AGT1*, *GDCSP*, *GDCST* and *GDCSH*, and thus, reducing photorespiration. To our knowledge, this concept has not been mentioned in other papers. Therefore, we hypothesize that ALA enhances photosynthesis and reduces photorespiration by promoting chlorophyll accumulation and alleviating the inhibition of photosynthetic electron transport chain, thus, alleviating drought stress. However, the specific photosynthetic indices related to this effect still merit further study and determination.

### 4.3 Effects of ALA on grapevine cell homeostasis under drought stress

There is a consensus that drought causes an imbalance in plant cell homeostasis. It has been reported that the foliar application of 3 µM ALA can not only increase the activities of SOD, CAT, GPX, GSH-Px, APX, DHAR, MDHAR, and GR but also increase the contents of AsA and GSH in cucumber (*Cucumis sativus* L.) under drought (Li et al., 2011). Similarly, we found that treatment with ALA significantly increased the activities of POD and SOD activities in ‘SM’ leaves over time. Furthermore, treatment with ALA upregulated antioxidant enzymes, such as *FSD*, *SODCP*, *APX*, *MDAR*, *DHAR*, and *GR*, under drought (**Figure 4C**), which could explain the increase in ALA antioxidant enzyme activity from a genetic perspective. In contrast to the results of the study described above, we found that the application of ALA reduced the levels of AsA and GSH in the metabolome (**Figures 4A** and **S10**). This could be owing to different results observed when different concentrations of ALA were sprayed in different types of drought mitigation. OA is a fundamental mechanism of drought adaptation in higher plants (Sanders and Arndt, 2012), Ji-Xuan et al. (2017) suggested that 50 mg L^-1^ of ALA could increase the contents of soluble proteins and the sugars and proline of Chinese ryegrass (*Leymus chinensis* [Trin.] Tzvel) to alleviate drought stress (Song et al., 2017). In the metabolome, we found that spraying ALA caused a sharp decrease in the contents of proline in grapevine leaves, which was caused by the downregulation of *PRO2*, *OAT*, and *PHR1.* In contrast to that study, the contents of raffinose, sorbitol, and trehalose all decreased after the application of ALA (**Figure 4D**) and alternatively, they were involved in the alleviation of drought by ALA. Autophagy plays an important role in the resistance of plants to drought stress (Tang and Bassham, 2022). Nevertheless, no study has reported that ALA alleviates drought stress by affecting autophagy. We found that many autophagy-related genes (ARGs) were highly expressed in the transcriptome of control *vs*. Dro comparison group, only one out of seven key ARGs that were identified were downregulated (**Table S28**). Among them, *ATG11* and *ATG2* were DEGs that play an important role in drought response. *ATG8F*, *ATG8I* and *ATG8C* were also expressed at high levels. After ALA treatment, the ARGs detected in the control *vs*. Dro comparison group were all downregulated (**Table S28**). Owing to the lack of direct experimental evidence, we anticipate that there will be subsequent studies on whether ALA affects plant drought tolerance by affecting autophagy. Nonetheless, our study interpreted the role of ALA in the maintenance of grapevine cell homeostasis under drought from the perspectives of genes and metabolites, and it was our goal that this study could provide some ideas for future studies on the mitigation of abiotic stress by ALA.

## 5 Conclusion

**Figure 5:**
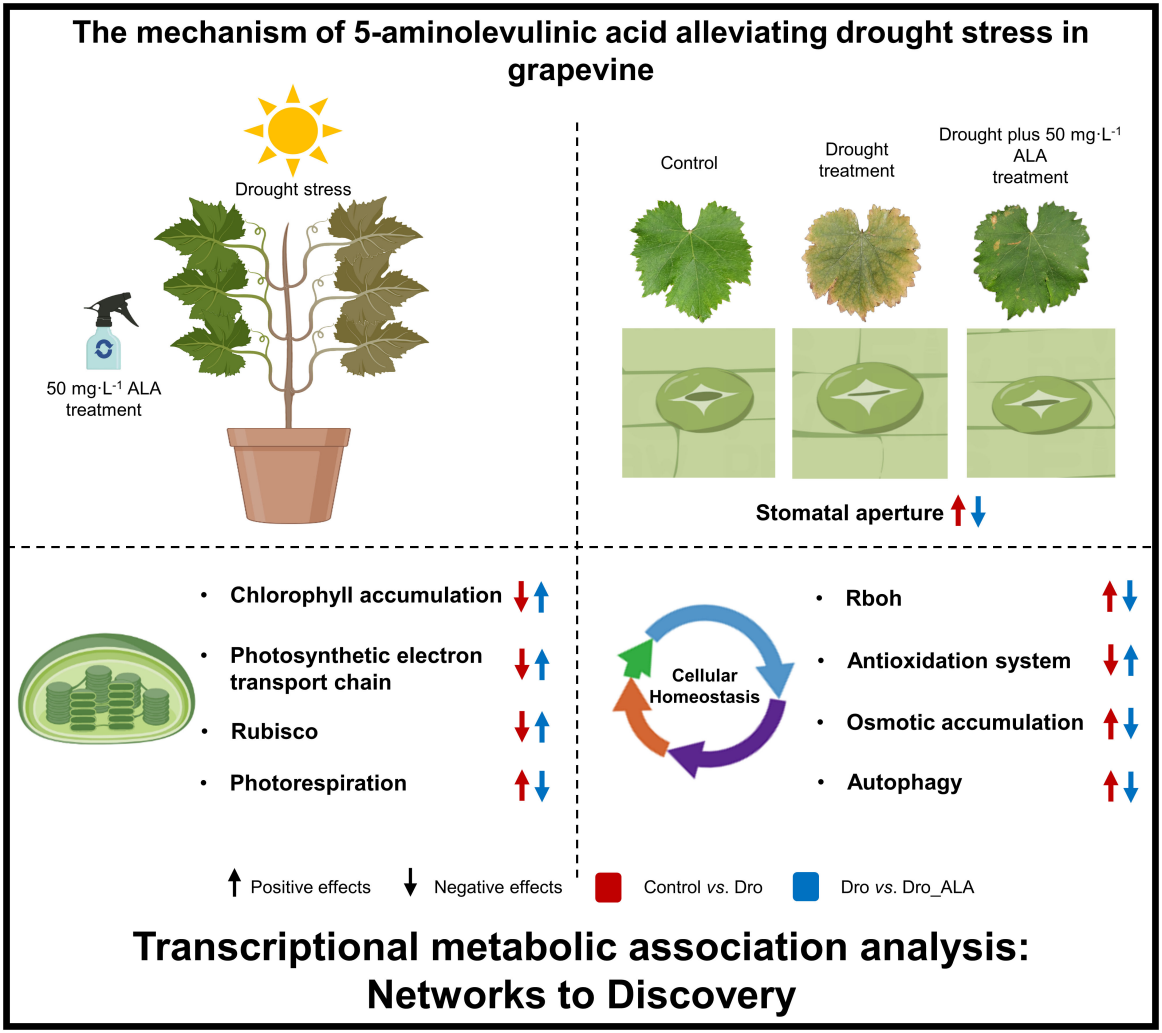
Regulation network of 5-aminolevulinic acid to mitigate drought stress in grapevine.

In this study, we confirmed that ALA can mitigate drought stress in grapevine. Stomatal movement, the chlorophyll biosynthetic pathway, metabolic pathway, photosynthetic mechanism, and cellular homeostasis constitute the basis of ALA’s regulatory network for alleviating drought stress. First, the application of ALA upregulated the genes related to ABA degradation, thus, inhibiting the stomatal closure induced by ABA, which alleviates the inhibition of drought on photosynthesis to some extent. The current data indicate that the DEGs associated with chlorophyll biosynthesis, such as *HEMA1*, *ALAD*, *PBGD*, *CPOX*, *PPOX, CHLH*, *CHLD*, *POR*, and *DVR*, were upregulated after ALA treatment, while the chlorophyll degradation genes *CLH*, *SGR*, *PPH*, and *PAO*, were downregulated, thus, mitigating the inhibition of chlorophyll accumulation caused by drought. ALA treatment also alleviated the inhibition of photosynthetic electron transport chain by drought. The genes related to Rubisco activity were upregulated by ALA, and photorespiration was attenuated. ALA down-regulated *RBOH*, thus, reducing the production of ROS and activating the antioxidant system, which changed the contents of antioxidants, such as tocopherol, ascorbate, and glutathione, to reduce the oxidative damage induced by ROS. *FSD* and *SODCP* were also restored to high levels of expression after the application of ALA. In addition, the decrease in the contents of betaine, raffinose, sorbitol and trehalose indicated that the degree of drought stress was reduced after the ALA had been applied. Therefore, this study explains the regulatory network of ALA to mitigate drought stress in grapevine (**Figure 5**) and provides a new concept to study the regulatory network of grapevine drought stress and apply plant growth regulators to alleviate other abiotic stresses.

## Data availability statement

The datasets presented in this study can be found in online repositories. The names of the repository/repositories and accession number(s) can be found below: https://www.ncbi.nlm.nih.gov/, PRJNA769649.

## Author contributions

YY wrote the original draft of the manuscript, managed and formalized the data. YY, JX, XF and HJ conducted the experiment. YY and YL carried out statistical analysis. JF and SL provided funds. XW, MG, SL, YP and XF polished the manuscript. All authors contributed to the article and approved the submitted version.
