# Supplementary material for: Drought stress in ‘Shine Muscat’ grapevine: Consequences and a novel mitigation strategy–5-aminolevulinic acid
Source: Front Plant Sci. 2023 Mar 15;14:1129114. doi: 10.3389/fpls.2023.1129114 (PMC10061586; doi:10.3389/fpls.2023.1129114)
Supplement: Supplementary file 1 [file DataSheet_1.docx]

Supplementary Figures

Drought stress in ‘Shine Muscat’ grapevine： consequences and a novel mitigation strategy—5-aminolevulinic acid

**Yuxian Yang^a,b#^, Jiaxin Xia^a,b#^, Xiang Fang^b,c#^, Haoran Jia^a,b^, Xicheng Wang^d^, Yiling Lin^a,b^, Siyu Liu^a,b^, Mengqing Ge^a,b^, Yunfeng Pu^e^, Jinggui Fang^a,b*^, Lingfei Shangguan^a,b*^**

*** Correspondence:** Jinggui Fang, E-mail: 2013151@njau.edu.cn;

Lingfei Shangguan, E-mail: shangguanlf@njau.edu.cn


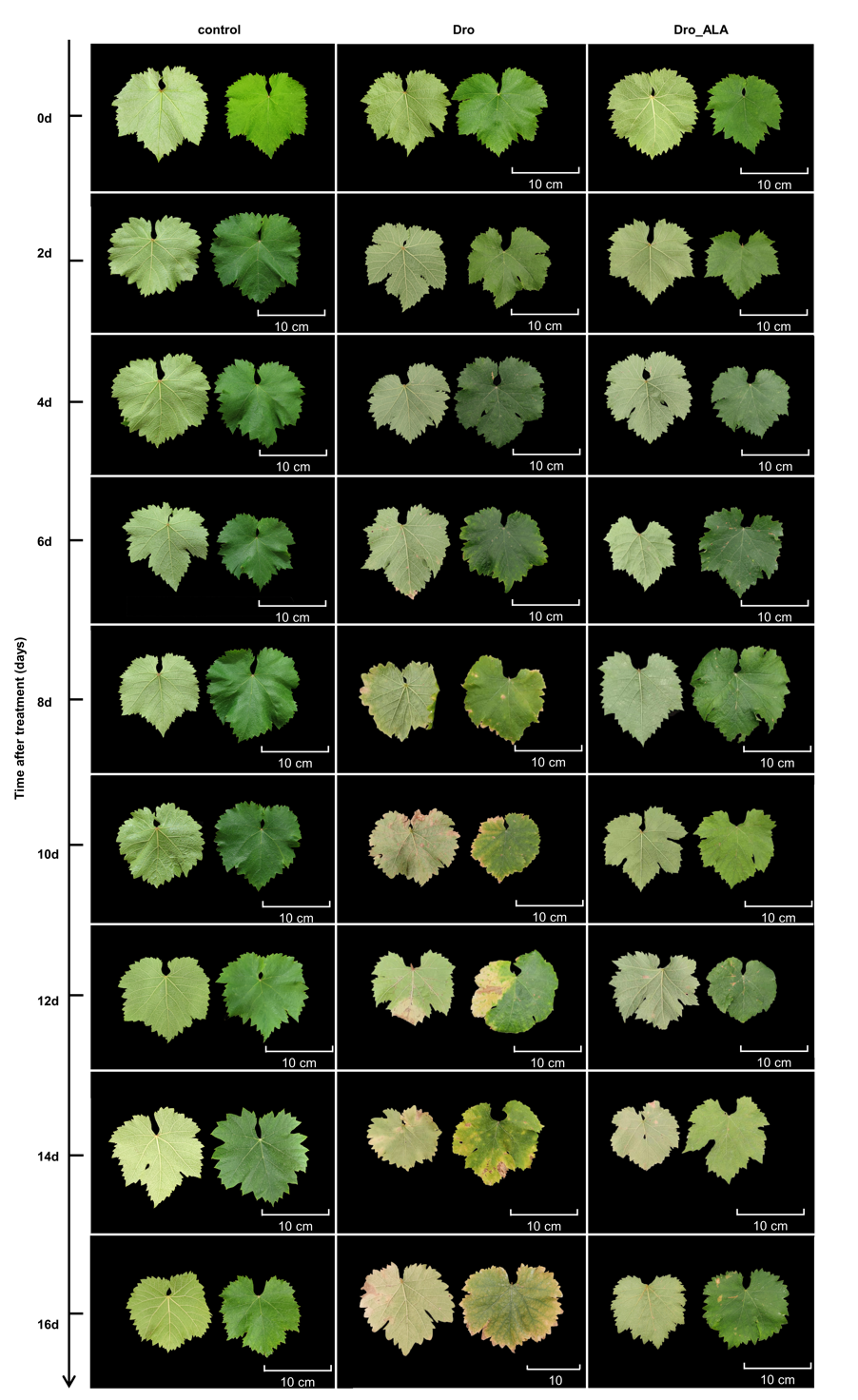


**Fig. S1. Leaf phenotype observation.** Leaf phenotypic changes in control, drought treatment (Dro), and drought plus 5-aminolevulinic acid (Dro_ALA) treatment. The surfaces of leaves began to wilt on the 4^th^ day after drought treatment. The obvious symptoms of drought appeared on the 10^th^ day. Dro_ALA leaves showed only mild symptoms of drought stress. (Note: d represents day.)


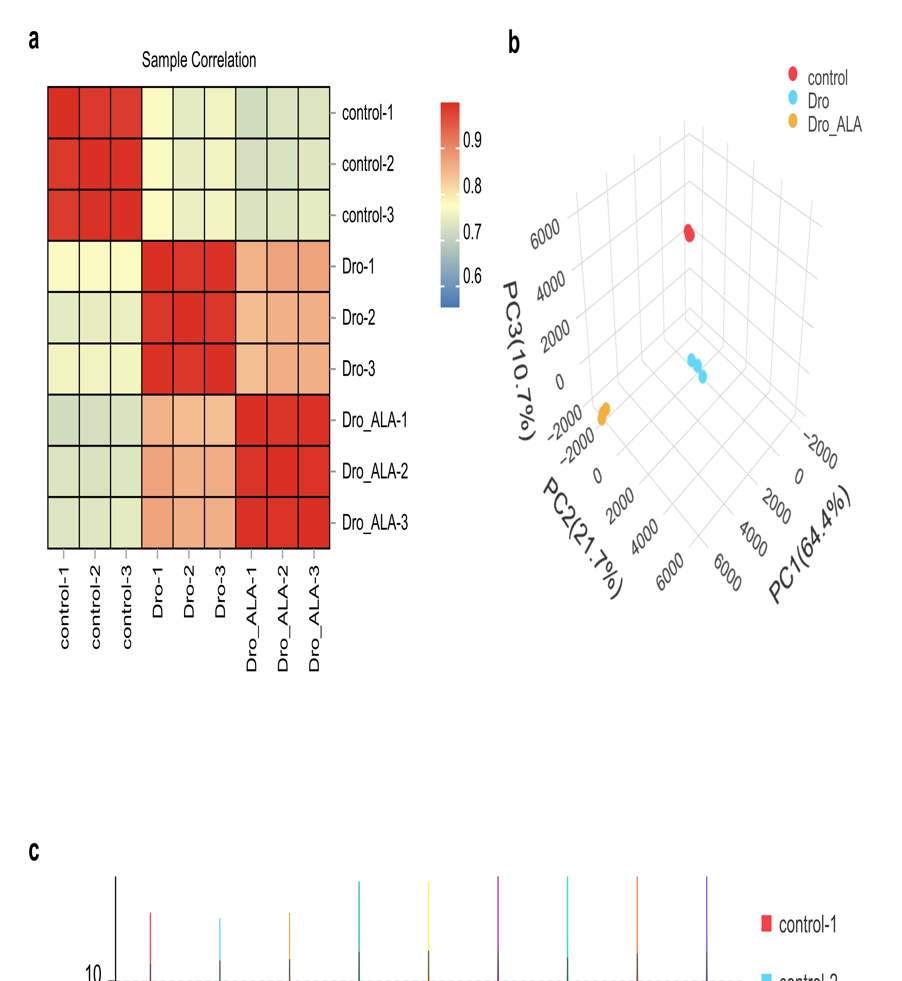


**Fig. S2. Analysis of the relationship between RNA-seq samples.** (a) Correlation between comparisons groups in RNA-seq data. The correlation value of each sample is indicated in the figure. (b) PCA analysis between samples. Principal Component Analysis (PCA) based on gene expression information. (c) Violin diagram of RNA-Seq data. Represents the abundance expression of genes, which can show the data density at any location.


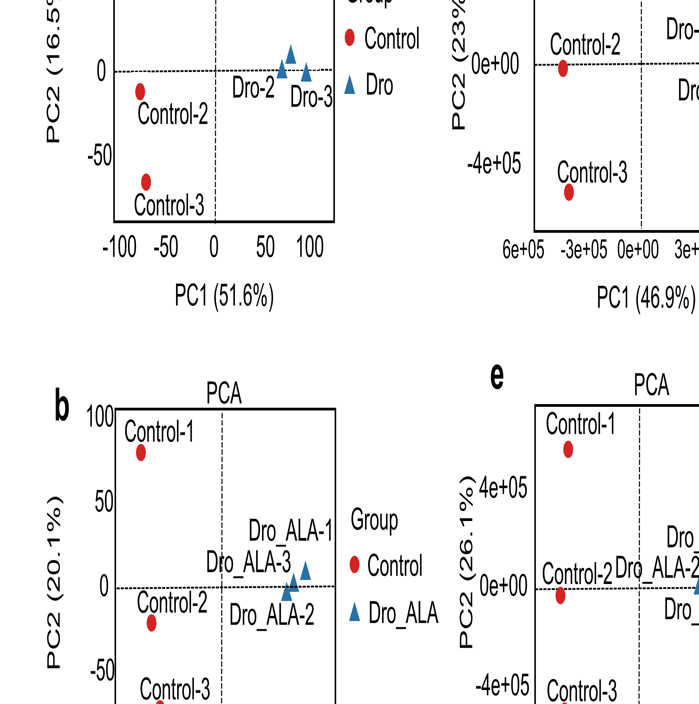


**Fig. S3. PCA score of test samples and quality control samples in the metabolome data.** (a) Positive ions detected in the control and Dro comparison groups. (b) Positive ions detected in the control and Dro_ALA comparison groups. (c) Positive ions detected in the Dro and Dro_ALA comparison groups. (d) Negative ions detected in the control and Dro comparison groups. (e) Negative ions detected in the control and Dro_ALA comparison groups. (f) Negative ions detected in the Dro and Dro_ALA comparison group. (Note: Dro represents drought treatment; Dro_ALA represents drought plus 5-aminolevulinic acid treatment.)


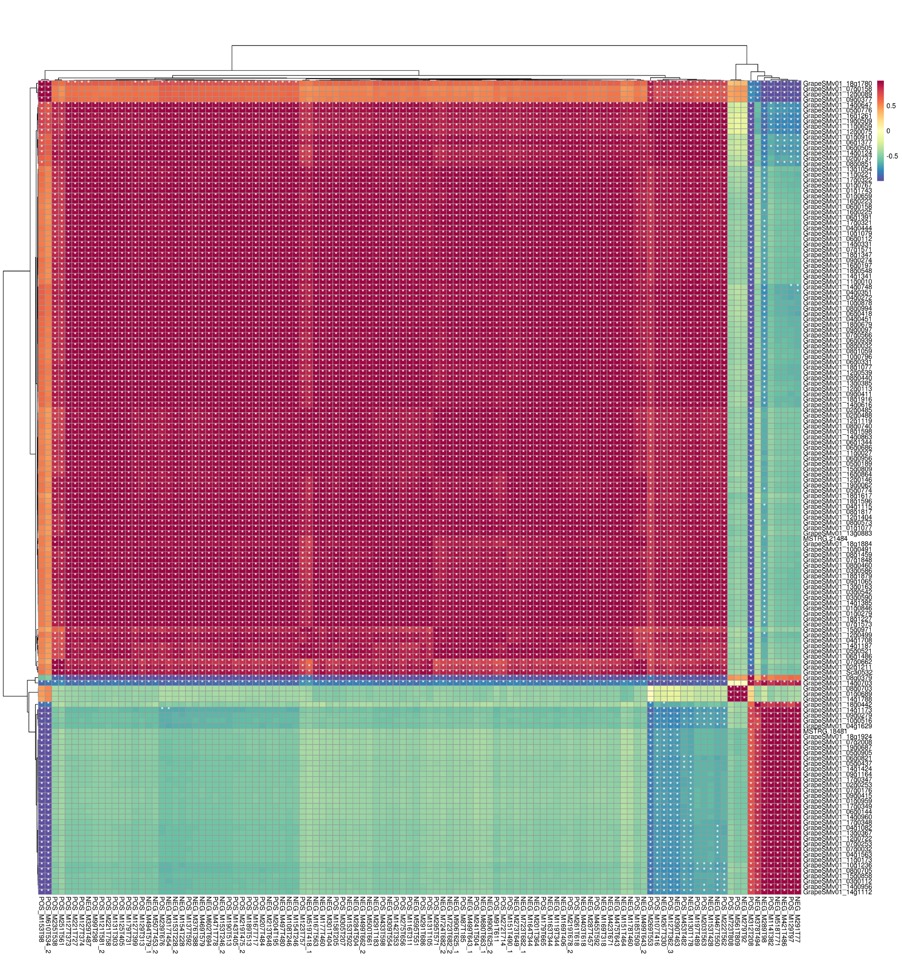
 **Fig. S4. Correlation among comparison groups based on the top 250 DEGs and DAMs**. Heat map of the correlations between the top 250 DEGs and DAMs. Pearson's coefficient of gene expression and metabolite abundance was used to evaluate the correlation between genes and metabolites.


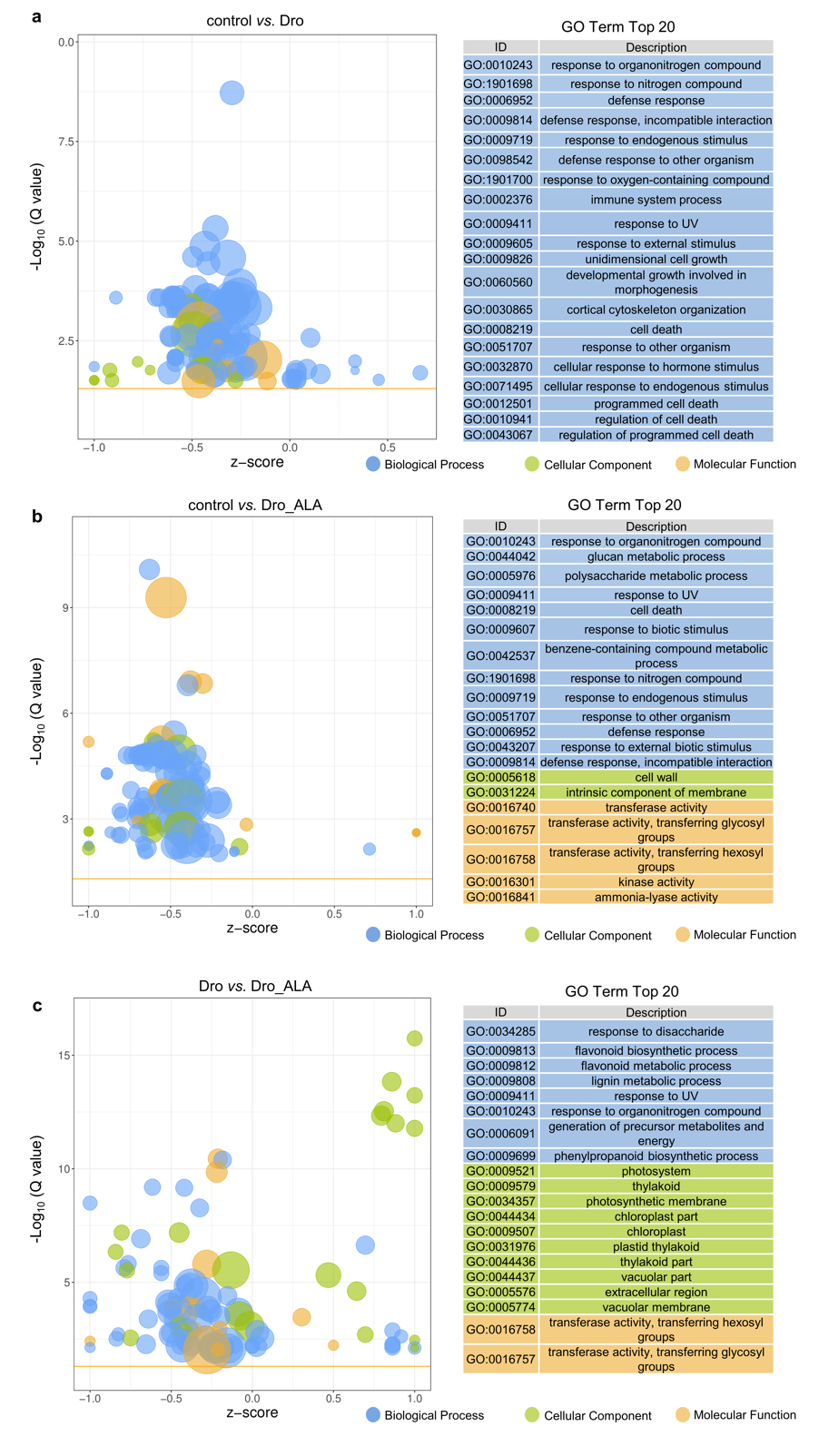


**Fig. S5. Transcriptome GO analysis.** (a) Top 20 GO terms of control *vs.* Dro, (b) control *vs.* Dro_ALA, (c) and Dro *vs.* Dro_ALA groups. (Note: Dro represents drought treatment; Dro_ALA represents drought plus 5-aminolevulinic acid treatment.)


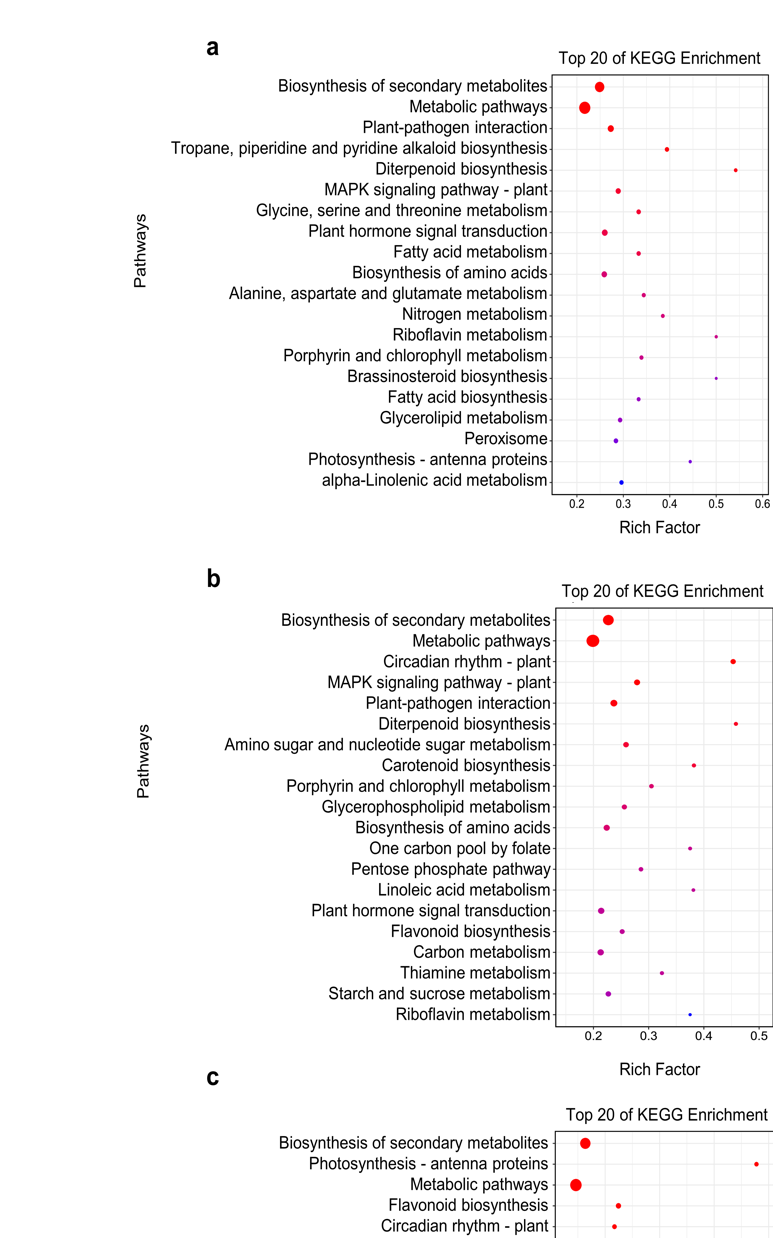


**Fig. S6. Transcriptome KEGG analysis.** (a) Top 20 pathways of KEGG enrichment of control *vs.* Dro, (b) control *vs.* Dro_ALA, (c) and Dro *vs.* Dro_ALA groups. (Note: Dro represents drought treatment; Dro_ALA represents drought plus 5-aminolevulinic acid treatment.)


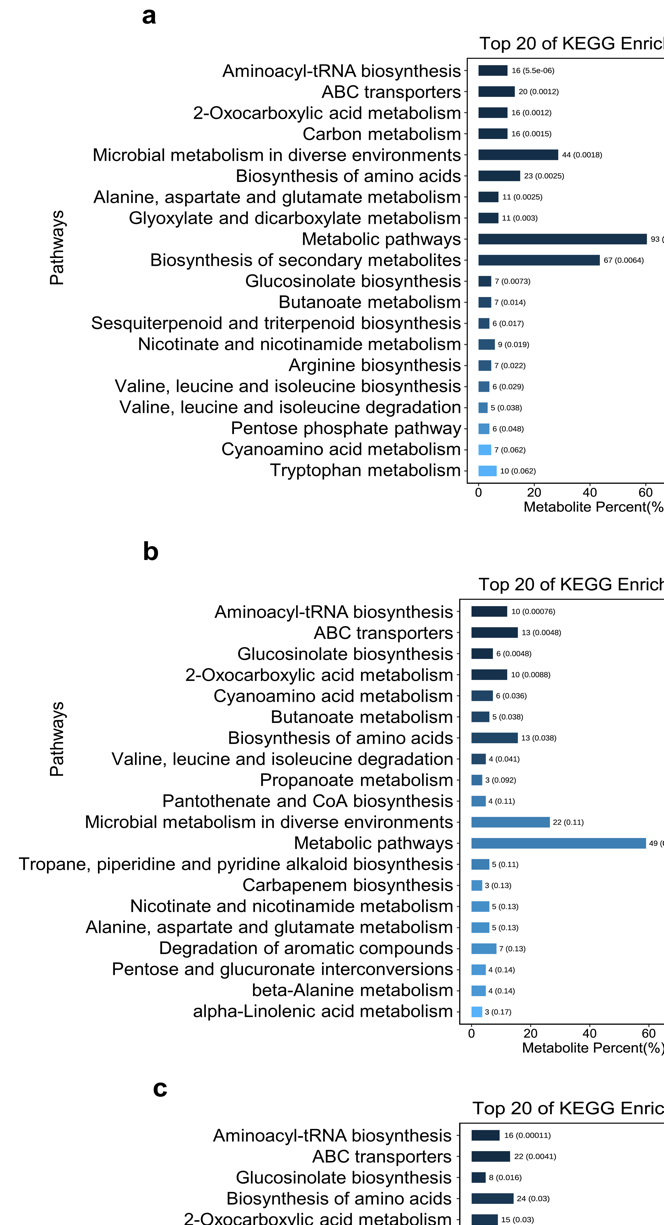


**Fig. S7. Metabolome KEGG analysis.** (a) Top 20 pathways of KEGG enrichment of control *vs.* Dro (b) control *vs.* Dro_ALA, and Dro *vs.* Dro_ALA groups. (Note: Dro represents drought treatment; Dro_ALA represents drought plus 5-aminolevulinic acid treatment.)


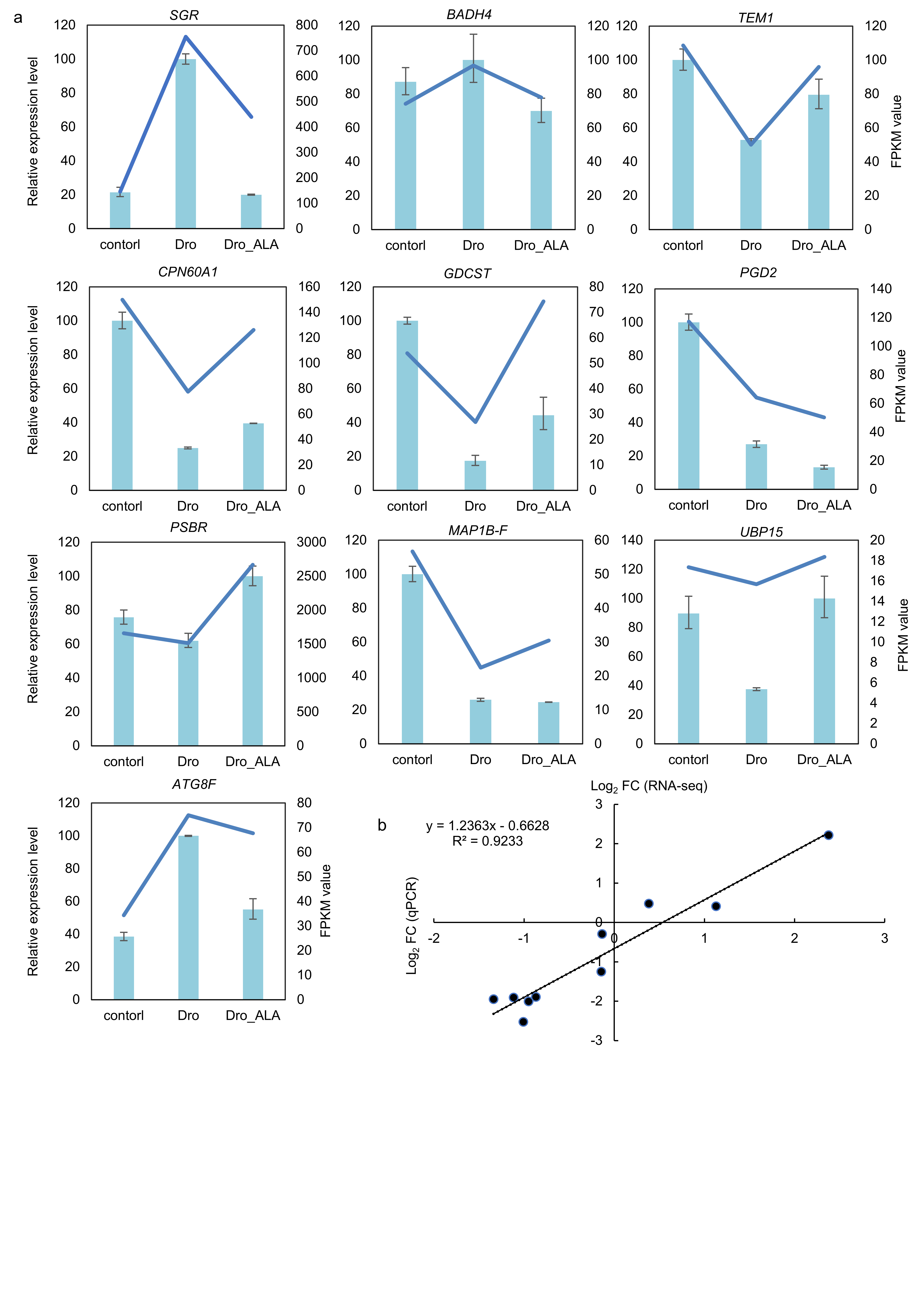


**Fig. S8. Validation of RNA-seq by RT-qPCR.** (a) The bar chart and the main ordinate represent the relative expression level of RT-qPCR, while the broken line chart and the secondary ordinate represent the FPKM value of RNA-seq. (b) Scatter plot of correlation between RNA-Seq and qPCR. (Note: Dro represents drought treatment; Dro_ALA represents drought plus 5-aminolevulinic acid treatment.


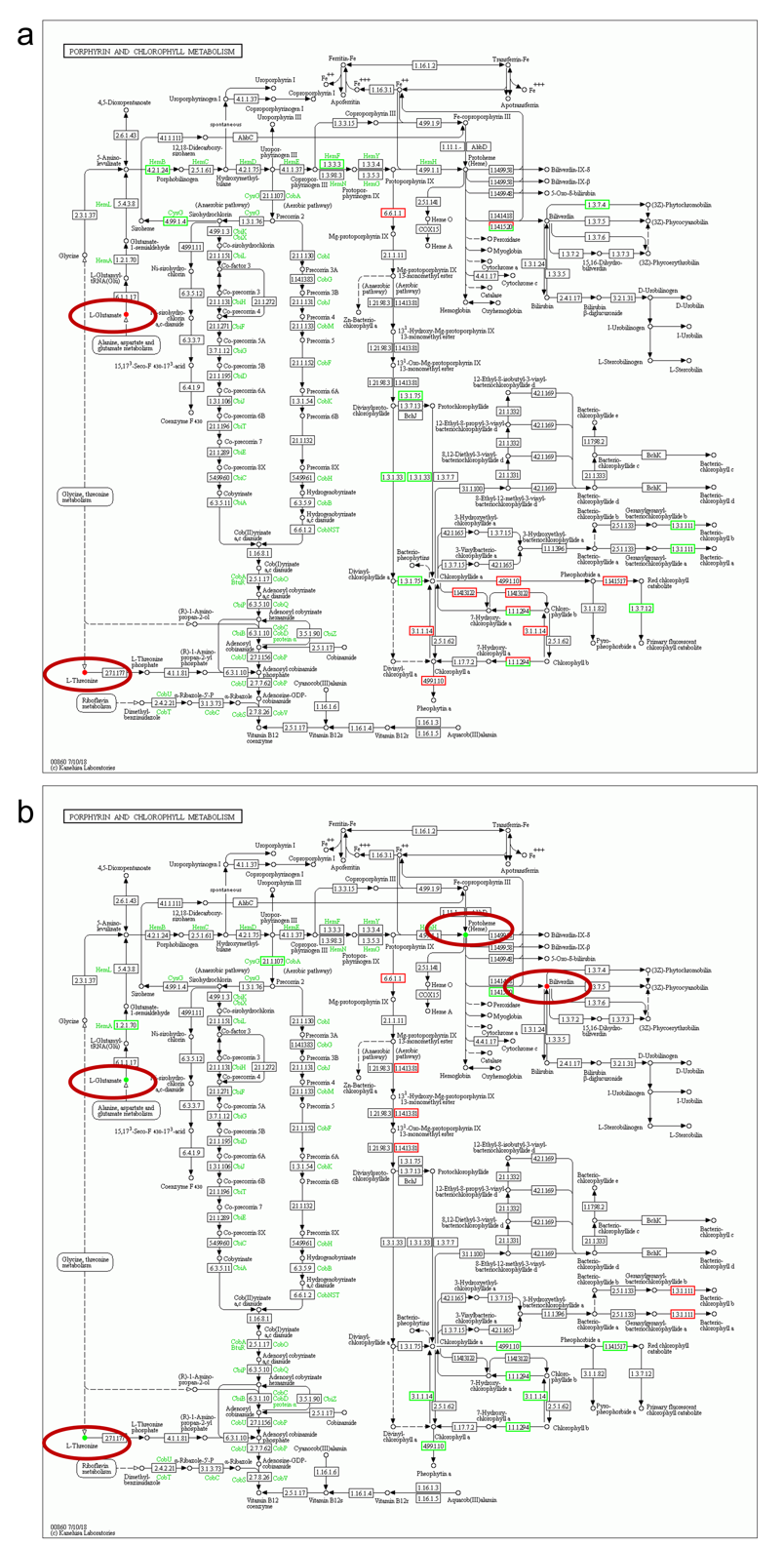


**Fig. S9. KEGG annotation of porphyrin and chlorophyll metabolic pathway in transcriptome and metabolome association analysis.** (a) control *vs*. Dro (b) Dro vs. Dro_ALA


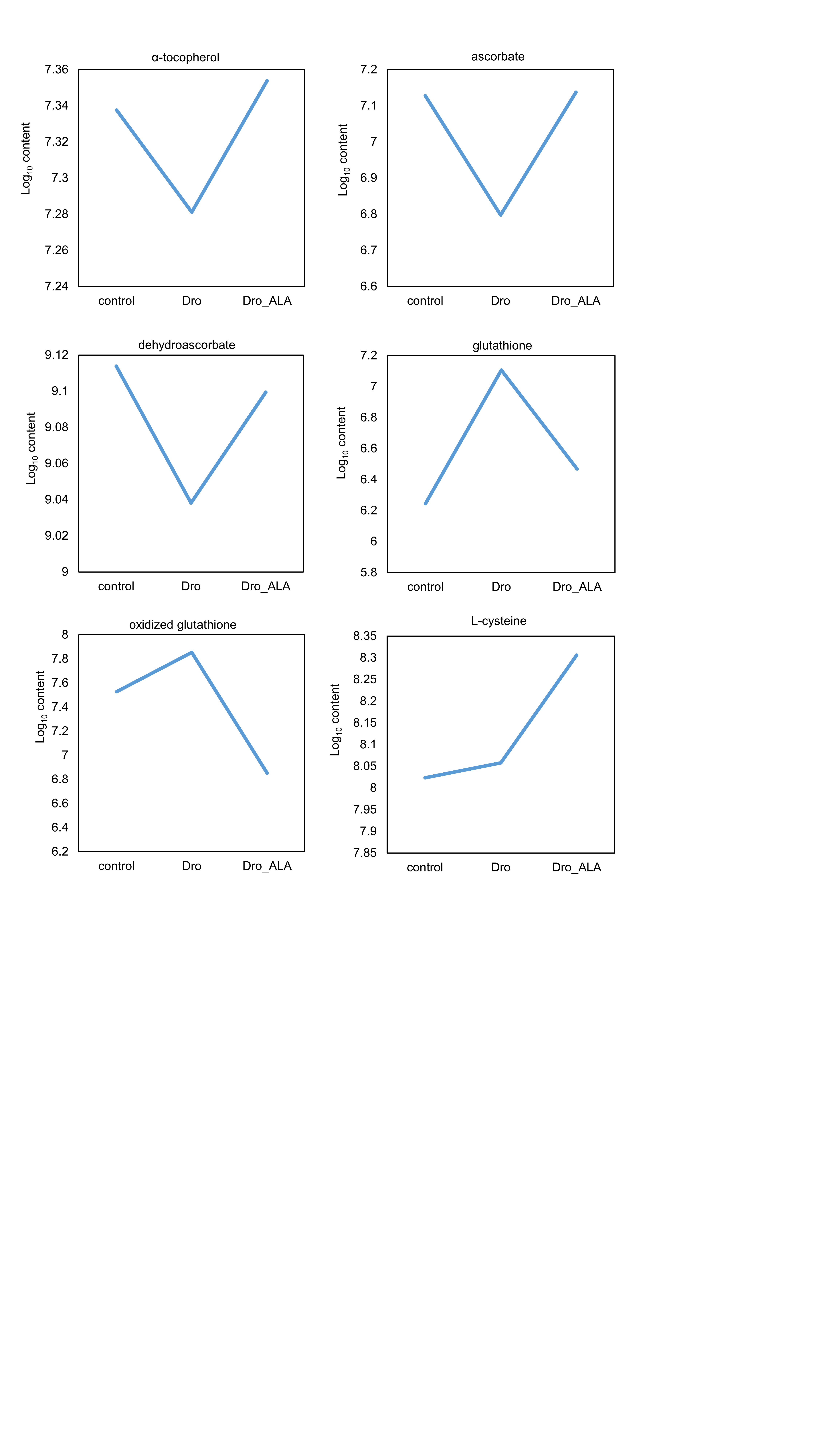


**Fig. S10. DAM changes of important antioxidant system after drought and drought plus ALA treatment.** Note: Dro represents drought treatment; Dro_ALA represents drought plus 5-aminolevulinic acid treatment.
